# A world free of malaria: It is time for Africa to actively champion and take leadership of elimination and eradication strategies

**DOI:** 10.4314/ahs.v22i4.68

**Published:** 2022-12

**Authors:** Chinedu Ogbonnia Egwu, Chinyere Aloke, Jennifer Chukwu, Anthony Agwu, Esther Alum, Ioannis Tsamesidis, Patrick M Aja, Christian E Offor, Nwogo Ajuka Obasi

**Affiliations:** 1 Medical Biochemistry Department, College of Medicine, Alex-Ekwueme Federal University Ndufu-Alike Ikwo, P.M.B. 1010 Ebonyi State, Nigeria; 2 Protein Structure-Function and Research Unit, School of Molecular and Cell Biology, Faculty of Science, University of the Witwatersrand, Braamfontein, Johannesburg 2050, South Africa; 3 World Health Organization, United Nations House Plot 617/618 Central Area District PMB 2861 Abuja, Nigeria; 4 Biochemistry Department, Ebonyi State University Abakaliki, P.M.B. 053 Ebonyi State Nigeria; 5 Department of Prosthodontics, School of Dentistry, Faculty of Health Sciences, Aristotle University of Thessaloniki 54124 Greece

## Abstract

The global burden of malaria seems unabated. Africa carries the greatest burden accounting for over 95% of the annual cases of malaria. For the vision of a world free of malaria by Global Technical Strategy to be achieved, Africa must take up the stakeholder's role. It is therefore imperative that Africa rises up to the challenge of malaria and champion the fight against it. The fight against malaria may just be a futile or mere academic venture if Africans are not directly and fully involved. This work reviews the roles playable by Africans in order to curb the malaria in Africa and the world at large.

## Introduction

Malaria is a vector-transmitted disease that has continually plagued humankind for years. Five species of the malaria parasite-*Plasmodium falciparum, Plasmodium vivax, Plasmodium ovale, Plasmodium malariae*, and *Plasmodium knowlesi* are known to cause malaria in human; however, *P. falciparum* is the most prevalent and deadly^1^. *P. falciparum* is the most dominant species in Africa, which accounts for majority of the malaria morbidity and mortality^2^. *P. ovale* is primarily concentrated in sub-Saharan Africa and islands in the western Pacific 1, while *P. vivax* is mainly found in Asia, Latin America and in some parts of Africa^3^–^5^, responsible for 3% of the global malaria burden. Lastly, *P. knowlesi* is concentrated in Southeast Asia^6^, while *P. malariae* has a wide global distribution, being found in South America, Asia, and Africa, but not as prevalent as *P. falciparum*^7^. *P. falciparum* and *P. vivax* are the most threatening species^2^. Malaria is a major cause of poverty as much as it is caused by poverty 8. It has a geographical distribution that has been classified based on its prevalence from spleen rate surveys as follows: holoendemic (> 75%), hyperendemic (51–75%), mesoendemic (11–50%) and hypoendemic (< 10%), when ascertained in children aged 2–9-year^9^. This endemicity is largely affected by the average weather condition of the region at a given period of the year.

The World Health Qrganization (WHO) estimated a global prevalence of 241 million cases in 2020 where sub-Saharan Africa once again took the top spot with more than 95 % of the global burden 2. It is worrisome to note that the control of malaria has stalled since 2014^10^,^11^, the cases increased by 14 million between 2019 and 2020 2 and this calls for a doubling of efforts or a paradigm shift by all stakeholders , especially Africans who are at the epicentre, to curb the ugly trend. According to Dr Pedro Alonso, a former Director of the Global Malaria Programme ‘Progress in malaria control, Africa is crucial to the attainment of the goals of the Global Technical Strategy”^12^. Due to the huge global malaria burden carried by Africa, especially sub-Saharan Africa, this article seeks to review the role that Africa can play to achieve a world free of malaria.

## The African Malaria Infection Context

A lot of factors affect the level of malaria infection across the malaria endemic regions. Such factors include but not limited to age; immunity; genetic diversity of the host; genetic diversity of malaria parasites and the regional climatic conditions 13–16.

### Climate

The climatic condition of a region is an important factor that affects the transmission and level of malaria infection. It is the most significant factor that affects the intensity of parasite transmission^17^. It is also important in planning and optimizing malaria strategies. The most important climatic conditions that have been reported to affect malaria parasite transmission are temperature and rainfall, with varying degrees of effect 18–20. The sub-Saharan Africa, where the burden is highest, has two major seasons annually- dry and rainy season, even though some areas may have double-peaked rainy season. During the rainy season, a lot of stagnant waters are collected and these serve as breeding grounds for the mosquitoes. The transmission of the parasite coincides with the spread of the rainy months in the different sub-Saharan African states^21^–^23^. This incidence heightens towards the end of the rainy season, as stagnation of water increases because of low runoffs. Mathematical models have revealed that the optimum climatic conditions for peak *P. falciparum* transmission are: temperature of 30–33°C and a daily rainfall in the range of 15–17 mm 24–26, which coincides with peak periods of transmission in Africa.

### Genetics of Africans and Development of Resistance to Antimalarials

The host genetic makeup is very essential in malaria infection as it accounts for approximately 25% of the total variability in malaria severity 27. There is a selection of several human survival mechanisms which include the genetic polymorphisms associated with the structure and function of the erythrocyte 28. Africans have unique genetics that manifest as ability to develop acquired immunity, haemoglobinopathies and/or enzyme deficiency. These unique characteristics enhance survival of Africans under malaria infection.

*P. falciparum* infection can lead to the development of acquired immunity in the host. The immunity is said to be mediated by immunoglobulin G (IgG) against variant surface antigens (VSA) from the parasite on the surface of parasitized erythrocytes^29^. This is often found in the adult population with well-developed immune system. Based on the level of protection enjoyed by the adult population in Africa due to the acquired immunity, it is proposed that the induction of such immunity in the vulnerable children population can drastically reduce malaria morbidity and mortality^13^. The ability of Africa, as a high transmission area to develop acquired immunity helps in the clearance of resistant strains of *P. falciparum* and consequently prevents the spread of resistance 14,15,30. This accounts for the historical slow development of resistance in Africa^31^–^33^. More so, the prevalence of the heterozygous sickle cell trait, a haemoglobinopathy in Africa has been reported to suppress intra-erythrocytic growth of *P. falciparum*^16^,^34^. Glucose-6-phosphate dehydrogenase (G6PD) deficiency found to be prevalent in African also confers protection from cerebral malaria, though associated with risk of anaemia 35,36. This is because G6PD is saddled with the production of NADPH which generates reduced glutathione (GSH) that prevents the erythrocytes from oxidative damage. Its lack or deficiency exposes the erythrocyte and the malaria parasite to a higher level of reactive oxygen species (ROS) that can kill the parasite.

Furthermore, the polymorphism in glycophorin receptors in Dantu genetic blood variant is of essence in protection against malaria in Africans. Glycophorin is a sialoglycoprotein of the membrane of red blood cells which plays a role in the invasion of the red blood cells by malaria parasites 37. Those with the rare Dantu blood variant have a higher surface tension that prevents the invasion of *P. falciparum* 38. As a result of peculiar African genetics, the region is characterized by a reduced time to development of resistance to antimalarials 33.

### Geographical spread and genetic diversity of malaria parasite

According to the WHO, *P. falciparum* accounts for about 99.7 % of the malaria cases in Africa 10 while *P. vivax* is concentrated in the Horn of Africa, comprising Djibouti, Eritrea, Ethiopia, Somalia, and Sudan 17, hence much of the focus on malaria control in Africa is on *P. falciparum*. There is growing prevalence of *P. vivax* in Africa where not less than 44 countries have reported it 39. These parasites have different genetic diversities. Because of the high transmission of the *P. falciparum* species in sub-Saharan Africa, the malaria burden is higher there. The genetic diversity of malaria parasites is important in the elimination and control strategies. With the recent increase in the prevalence of *P. vivax*, policies and programs of malaria should capture other species of malaria in order to have a holistic approach in the fight against malaria in the African region.

### Geographical spread and genetic diversity of Mosquitoes

The transmission of malaria parasite by mosquito is elevated in areas where the mosquitoes have lifespan long enough to allow for completion of malaria parasite lifecycle and also the mosquitoes human-biting habit. Examples of the female *Anopheles* mosquitoes responsible for *Plaasmodium* transmission are *A. gambiae, A. funestus*, *A. pharoensis. A. squamosis, A. coustani* and *A. ziemanni*. This is characteristic of the mosquitoes in Africa and invariably responsible for the huge malaria burden in Africa 30. The emergence of *A. stephensi* further complicates the issues. *A. Stephensi* is an Asian species that thrives in urban centres in Africa. It was responsible for a malaria outbreak in Djibouti, East Africa in 2012 40. Because of the threat that this species poses to malaria control, the WHO has called for a targeted approach in its elimination 40,41. The survival of these mosquitoes is enhanced by pools of stagnant water especially during the rainy season in Africa. More so, the feeding and resting behaviours of these mosquitoes enhance their vectorial capacity. These unique vector behaviours should be factored in the malaria elimination approach (insecticide application) 42.

## Gains made and challenges facing malaria control in Africa

**Figure 1:**
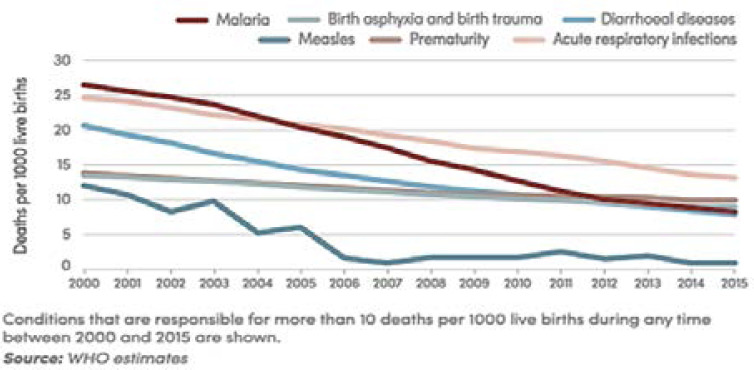
Malaria no longer number one cause of death in children under 5 in sub-Saharan Africa 10

Between 2000 and 2015, there was a commendable decline in malaria morbidity and mortality by 42% and 66% respectively. That made malaria to move from number one (17%) to number four (10%) cause of death in children under 5 years in sub-Saharan Africa 12 (Figure 1). According to Dr Matshidiso Moeti, WHO Regional Director for Africa ‘Malaria is no longer the leading cause of death among children in sub-Saharan Africa.” 12 (Figure 1).

### The Success Story

**Figure 2:**
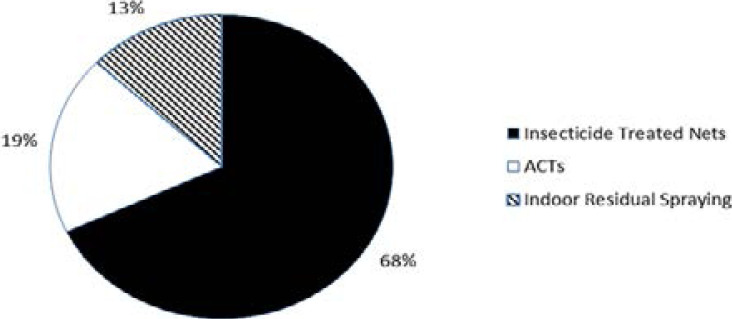
Percentage contributions of malaria control tools in the control of malaria between 2000–2015 45, The contributions are measured based on the prevention of malaria morbidity.

**Figure 3:**
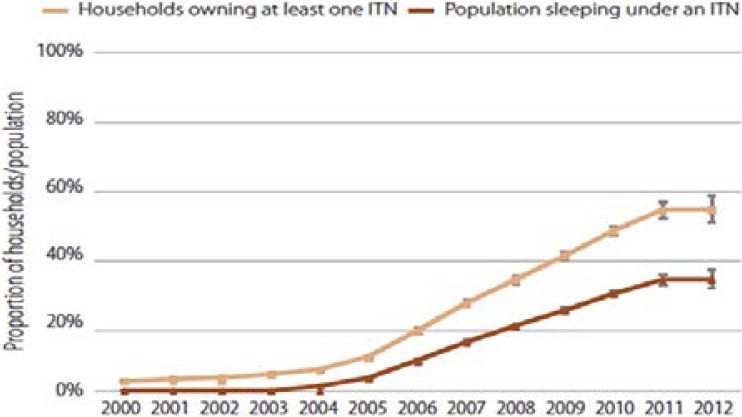
Distribution and use of insecticide treated nets. The trend shows a steady increase in the distribution and use of insecticide treated nets^47^

A lot of strategies has been used to tackle malaria head on in Africa, which could be preventive or non-preventive. Among the preventive strategies are: sleeping under insecticide treated nets, keeping the environment clean, mass literacy programs, use of outdoor/indoor insecticide sprays, use of mosquito repellants and use of prophylactics while non-preventive strategies involve mainly the use of artemisinin-based combination therapies (ACTs)^43^–^45^. According to the WHO report, the deployment of ACTs rose from 11 million in 2005 to 337 million in 2014, Africa benefiting 98% of the treatment course 12. The use of insecticide treated nets has been hailed as effective in preventing malaria prevalence 46. Statistically, the World Malaria report shows that the use of insecticide treated nets has played a significant role in the success story so far in curbing malaria by accounting for 68% of malaria morbidity prevented between 2000–2015 in Africa 45 (Figure 2). Within this period, the distribution and use of insecticide treated nets increased significantly (Figure 3). This effort was complemented by the massive use of ACTs (19%) and indoor residual spraying (IRS). It is therefore necessary to consolidate these interventions that have led to the overall success story between 2000–2015 45.

### The Vision 2030 for Africa

Considering the gains made so far and the challenges therein, African WHO member states have adopted a framework from 2016–2030, to guide them to achieving a malaria-free Africa 12. This vision is aligned to the goals of the Global Technical Strategy 48 for Malaria whose timeline is aligned to the UN 2030 Agenda for Sustainable Development. The objectives of the framework are:

i. Reducing malaria mortality rates by at least 90%;

ii. Reducing malaria case incidence by at least 90%;

iii. Eliminating malaria from at least 20 malaria-endemic countries;

iv. Preventing re-establishment of malaria in all Member States that are malaria-free.

## Challenges to Malaria Control in Africa

**Figure 4:**
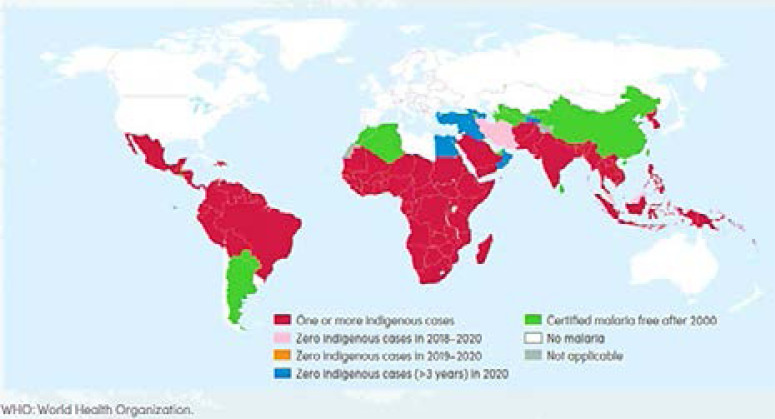
**Countries with indigenous cases in 2000 and their status by 2020.** Several countries have achieved great feats in the war against malaria via total elimination, which is in line with the Global Technical Strategy for malaria 2016 – 2030. China and El-Salvador have been certified malaria free by the WHO in 2021. Belize and Cabo Verde reported zero indigenous malaria cases in 2019 for the first time since 2000. While Timor-Leste reported zero indigenous malaria cases in 2018 and 2019 and had three indigenous cases in 2020, Iran and Malaysia have had zero malaria cases for three consecutive years. Note: Countries with zero indigenous malaria cases for at least the past 3 consecutive years are considered free. Africa as a unit still carry the highest burden^2^,^48^.

The gains made in malaria control seems to be gradually lost as a closer look at the morbidity figures shows that the reduction in prevalence has stalled 2. A lot of factors are working against the commendable malaria programs. These confounding factors are the reasons Africa remains the region with the highest burden with no significant progress in malaria prevalence (Figure 4).

### Access to medicine

The use of drugs is important for timely treatment of malaria. In Africa, access to drugs is usually complicated by a lot of confounding factors such as natural barriers (river, mountains etc.) which reduces accessibility to health facilities; corrupt officials who would rather divert free drugs for other purposes than give them to intended targets; poor orientation of the populace about the need for prompt treatment and the cost of the drugs especially ACTs in the face of underlying poverty 49,50. Limited access to antimalarials is arguably one of the contributors to the development of drug resistance 51. The importance of access to medicine cannot be underestimated as it has been reported that mass drug administration markedly reduces malaria in malaria hotspot villages 52–54. The standard of the healthcare system in Africa is generally below the recommended level 55. This is strongly correlated to malaria control. Thus, strengthening health systems can be an effective strategy for reducing malaria cases, mostly in malaria endemic countries 56.

### Fake drugs/counterfeiting

The proliferation of fake medicines in Africa is a public health crisis that has not been properly managed. Antimalarials could be substandard, degraded or falsified and can lead to treatment failure/resistance, death and financial losses resulting from a sub therapeutic exposure of the parasites to the drug 57. Fake drugs in Africa may actually be underreported as the WHO says that one in every ten drugs in developing countries are counterfeits 58. This underscores the gravity of the challenges at hand in Africa. The incessant availability of fake drugs can continually make the achievement of a world free of malaria unattainable and therefore has to be quickly and continually addressed.

### Poor diagnosis

Prompt malaria diagnosis is essential for effective treatment especially in preventing development of severe malaria and consequent death. Even though in sub-Saharan Africa, rapid diagnostic tests (RDTs) are becoming increasingly the most used method to test for malaria 59, presumptive approach is still common and a lot still needs to be done in the area of prompt diagnosis. Based on presumptive diagnoses, treatments are therefore made using signs and symptoms of malaria which may be misleading sometimes. More so, good diagnosis is impeded by poor quality of diagnostic tools such as RDTs and microscopy. Majority of the RDTs (>80%) are based on the detection of histidine-rich protein-2 (HRP2) 60. Mutations (gene deletions) of HRP2 in the malaria parasite has reduced the sensitivity of the RDTs 61. A false-negative result can probably be obtained due to the purchase and use of poor-quality RDTs and microscopy 62,63.

Malaria control and elimination is complicated by the presence of asymptomatic malaria population with low parasite density which serve as reservoirs for malaria transmission 14,16,64. Most malaria cases in Africa are treated at home and this gives room for a high level of erroneous treatment. Techniques that are noninvasive which can detect asymptomatic cases are recommended to aid effective diagnosis 64, hence ensuring effective treatment and reduced transmission. The WHO global malaria program recommends a written diagnostic policy and guideline in healthcare facilities geared towards making diagnosis a prerequisite for treatment 65.

### Abuse/ Misuse/Underuse of Malaria Arsenals

Because of the poor diagnosis and lack of awareness, some of the malaria control strategies are either abused or misused. Abuse is more common with the antimalarials. Oftentimes, there is little, poor or no diagnosis before malaria treatment and incompletion of treatment. This leads to poor parasite clearance, recrudescence and potential resistance development. The poor parasite clearance in patients increases malaria transmission as they serve as reservoirs for onward transmission to others 16. The insecticide treated nets are misapplied in some localities in Africa and sometimes under-utilized. Even though the distribution of insecticide treated nets (ITNs) has increased significantly, in some countries in Africa, their use in children under 5 is still below the recommended level and as low as <30% 66. Instead of using them to cover themselves at night, many people have resorted to using them as fishing nets, nursing seeds, fences in gardens and/or as football nets 46.

More so, there is low compliance to a lot of malaria programs that have been rolled out. For instance, according to Dr Matshidiso Moeti, WHO Regional Director for Africa, regardless of the recommendation of at least 3 doses of sulphadoxine pyrimethamine (IPTp-SP) as intermittent preventive treatment in Pregnancy (IPTp), only a paltry 19 % of eligible pregnant women complied in Africa in 2016 30.

### Resistance to Antimalarials and Insecticides

Historically, the development of resistance to antimalarials like the antifolates (sulfadoxine and pyrimethamine) and quinolines (chloroquine and mefloquine) originated from Southeast Asia and spread westwards to India and then to Africa 32. It is worrisome to note that resistance to artemisinin and its derivatives, confirmed by k13 gene mutation as molecular marker seems to have taken the same trend 31,32,67. Although K13 mutation, the main marker for artemisinin resistance, has been reported in Africa (e.g. Rwanda and Uganda) due to artemisinin exposure, there has not been a strong link to *P .falciparum* artemisinin resistance in Africa 68–71. However, caution must be exercised as artemisinin resistance is projected by a mathematical model to be in Africa not earlier than 2020 33. A widespread resistance has not been reported in the continent as of 2021. A widespread resistance to artemisinin as predicted would be a big threat to global war against malaria especially as the ACTs have been used as effective first-line therapy in malaria treatment. A malaria workshop held in Bangkok on December 1^st^ 2014 recommended the urgent tackling of the antimalarial resistance in Southeast Asia before it spreads to Africa 32.

Recently, mosquito resistance to the commonly used classes of insecticide, pyrethroids, organochlorines, carbamates and organophosphates has been reported 59. The pyrethroids are the class currently recommended by the WHO for use in ITNs 59. Mosquito resistance development to at least one insecticide has been reported in ≥ 64 malaria endemic countries globally and the trends is rising in Africa, Southeast Asia and Latin America 72,73. This poses a threat to the actualization of the malaria vision of 2030 because ITNs have been noted as major contributors in the success registered from 2000 to 2015 12,45. Insecticide resistance has not been directly linked to loss of protection by the bed-nets. However, the creation of holes in the bed nets leads to reduction in protection with or without insecticide resistance 74.

### Lack of sustainability of project funding

**Figure 5:**
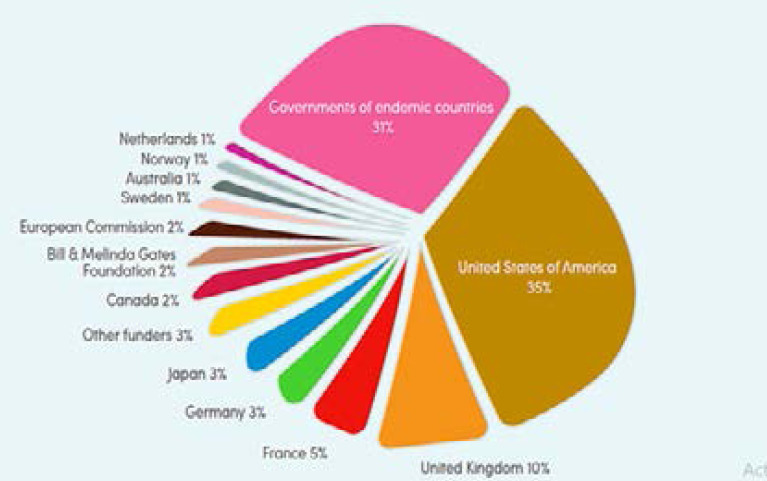
Funding for malaria control and elimination, 2010–2019 (% of total funding), by source of funds (constant 2019 US$). The records show that majority of the funding in endemic regions come externally 10.

A lot of commendable malaria projects with timelines are rolled out regularly, but once the funding period elapses, all the gains made from such projects are lost 75. There are several causes of non-sustainability in funding: paucity of fund; complacency by stakeholders; war or disaster; intentional cessation of control activities and/or lack of cooperation from host communities 76. According to Dr Matshidiso Moeti, WHO Regional Director for Africa, financing of malaria projects is not matching the pace of the intended aspirational level in the Global Technical Strategy of 2016–2030 30. This stalls a lot of laudable malaria projects in the African region, consequently working against making Africa and the world at large, free of malaria. More so, majority of the funding for malaria projects in endemic countries come externally 10 (Figure 5), so a lot has to be done in this regard to improve sustainability of these projects. Malaria projects like “Roll back Malaria”, and “Malaria Consortium” suffered serious setbacks in the fight against malaria due to several factors 77. Proper project monitoring is essential to ensure completion of malaria projects.

### Poor state of healthcare infrastructure

Although efforts are ongoing in improving the healthcare systems in Africa, they are still below the level recommended by the WHO 59. Some healthcare facilities in Africa are not well equipped, or in comatose. The government at different levels should revive the healthcare facilities for easy access to treatment by patients.

### Miscellaneous Challenges

Other challenges that frustrate actualization of Africa free malaria are: favorable climate for mosquito growth, poverty, literacy level of the populace, poor building structures that encourage indoor mosquito bites, insurgence that render people homeless and vulnerable to mosquito bites 78.

## Stakeholders' roles in malaria control in Africa

Every African has a stake in the burden of malaria. One is either infected or affected. Whichever spot one takes, the impact is huge. Every African is a stakeholder in the fight against malaria; therefore all hands must be on deck to win the war. Everybody should ask himself/herself the big question, **“What can I do as a stakeholder to win the war against malaria?”** Until this question is objectively answered by every stakeholder, the quest to win the war against malaria may be a mirage or impossible.

### The government

The government can come to the discussion table with industries, agencies and parastatals to fashion out the role each could play in raising awareness and funding malaria projects in Africa. In 2012, the Tanzanian Prime Minister, Hon Mizengo Pinda launched a Malaria Safe program and reached out to 32 Tanzanian companies for participation in the program 79. In Nigeria, the Federal Ministry of Health came up with the National Malaria strategic plan (2014–2020) to eliminate malaria in Nigeria 80. The malaria workshop held in Bangkok on December 1 2014 recommends the involvement of heads of governments to promote the multi-sectorial approach against malaria 32 and according to the WHO, there is need for African governments to complement international funding agencies in order to win the war against malaria 59. It is commendable to note that in a 2014-outlook report, Ghanaian government's contribution in research and development (R and D) was 68%, the highest posted by any African country 81. To surmount the health challenges, investment in R and D is necessary to achieve the desired global impact 82.The African governments should therefore provide enabling platforms for the actualization of set malaria goals by organizing the different teams, funding relevant malaria projects, equipping the health facilities and provision of infrastructure. The African government should show a strong political will and emulate countries like China who have achieved malaria-free status through several laudable malaria elimination initiatives 2.

### The academia /researchers

This group stands as a bridge to the gap in actualizing the vision 2030 of global malaria strategy. The role of research and development for health in eradicating poverty and economic growth cannot be under-estimated 82,83. The roles of researchers include but not limited to doing effective research to continually find ways to control malaria such as antimalarials and insecticides; demystifying the meaning of malaria; communicating results or findings to the masses and/or advising governmental and non-governmental agencies on approaches to adopt. Currently, a lot of research has been done on malaria, but it is pathetic to note that majority of these studies are done by non-Africans or outside Africa. Research in Africa has historically faced the challenge of poor funding, inadequate mentorship etc., which adversely affects research in Africa 84. Truly, the academia in Africa need to wake up to the realities on ground and it is high time that African scientists championed the malaria research and tell the malaria story themselves.

The researchers in Africa should form a formidable network between them and the industries so that the dynamics in malaria can well be taken care of.

### The media

No doubt, there are several commendable malaria programs that have been or yet to be rolled out. Regrettably, some of the intended targets do not benefit from some of the programs as a result of information gap. Sensitization of the masses through the right media channel is therefore important to making people understand the benefits from such projects. Such media channels could be radio, television, print media or social media. It is commendable to note that some media houses have slots for malaria programs and their impacts on reducing malaria prevalence have been significant 85,86. It is therefore recommended that more media houses should embark on sensitization with or without support from sponsors in order to spread vital information about malaria to the populace. Such information may include but not limited to effective use of insecticide treated nets; need for pregnant women to subscribe to intermittent preventive treatment; intervention approaches in cases of emergency and hosting panelists on sensitive malaria subjects. Frantic efforts should be made to prevent misinformation from the media houses.

**Figure 6:**
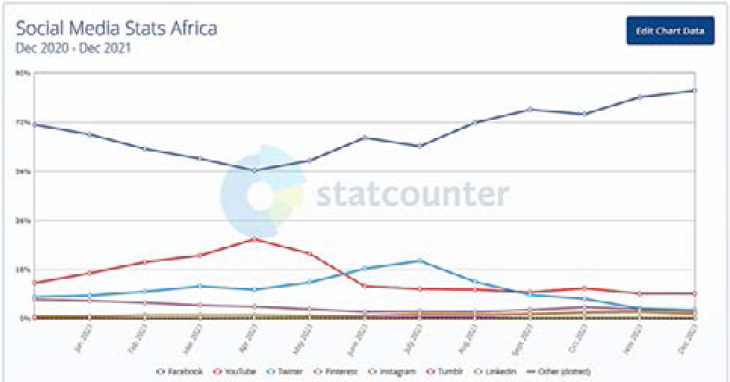
Social Media use in Africa. Facebook is the most used social media in Africa^87^

Recently, the use of the social media has been on the rise. According to the recent global statcounter, Facebook accounts for at least 80 % of the total social media usage in Africa 87 (Figure 6). African bloggers, who often have a lot of followers on Facebook, can tap into this followership to champion the course of cascading relevant malaria programs. WhatsApp and other social media platforms that are readily used can also be handy in disseminating vital malaria information.

The print media is another platform to disseminate malaria programs. With the e-versions of newspapers, communication of programs to the target audience has become easier and cheaper.

### The Celebrities

The fan-base of most celebrities is rapidly growing in Africa. The celebrities could be sportsmen and women, entertainers etc. This provides a readily accessible platform for fans and others to get tips about malaria and other diseases that are preventable and treatable in Africa. The celebrities are followed by their fans on the social media and on the field of play. Therefore, the African or non-African celebrities can play a huge role by passively or actively disseminating the information about malaria. The voice of Yvonne Chaka Chaka of South Africa has been noticeable in this context. As a singer, she has been championing the fight against diseases and she has risen to become a UNICEF goodwill ambassador for malaria in Africa. Innocent Idibia, a popular musician from Nigeria has also used his voice through several malaria campaign programs. Novelty matches can be organized and dedicated to raise awareness or fund for the malaria projects in Africa as was exemplified in 2009 in a game between Ghana and Mali 79.

### Corporate organizations

For sustainable fight against malaria in Africa, the corporate organizations in Africa also have to play a critical role through corporate social responsibilities. They can do this by ensuring regular diagnosis and treatment of their staff and donation of insecticide treated nets to the public. They can also fund research by the academia in order to combat the ever-dynamic disease. The good news is that some corporate bodies in Africa are already doing so. Said Salim Bahresa and Co (SSB), an East African flour mill in Tanzania is noted for donating insecticide treated nets, rapid diagnostic kits and drugs to her employees since 2008, and since 2006, Mr Zake of Standard Chartered Banks has been funding the distribution of nets to about 17 sub-Saharan African countries 79. ExxonMobil and Coca-Cola Africa Foundation have also been actively involved in the distribution of insecticide treated nets in Africa. Beyond theprovision of nets, ExxonMobile has commendably been involved in building health facilities and training and retraining of health personnel in Nigeria^88^.

Although some companies have keyed into this noble project, many are still left behind. It is therefore recommended that companies especially the pharmaceuticals should, as a matter of necessity, incorporate research funding and combating epidemics like malaria as a core component of their social responsibility. More so, funding of research and development has been abysmally poor in Africa. Except for South Africa where private businesses contribute up 40% in R and D, in other countries, they contribute poorly, as low as 0.1% 81. It is therefore pertinent that scientific research is awakened in Africa via funding as a corporate social responsibility from companies who will indirectly benefit from such research.

### The general public

The generality of Africans is supposedly involved in the fight against the scourge of malaria rightly or wrongly. It is therefore pertinent that everybody knows his/her role in the fight. The most important approach to malaria control is prevention. This could be either at the level of preventing mosquito breeding or mosquito bites to prevent transmission. The masses can simply prevent breeding of the mosquitoes by keeping the environment clean, clearing/avoiding pools of stagnant and exposed water bodies. They can also consistently sleep under mosquito nets as prescribed by the WHO. Each African should champion malaria advocacy programs at the family level, which will be a formidable bottom-up approach in the fight against malaria.

## Conclusion

Malaria is not abating and this is indeed a worrisome trend. The current situation is surmountable if the entire key players are actively involved. The role of the aforementioned stakeholders cannot be overemphasized. Africa has to rise up and start telling their stories themselves. Foreign aids are inevitable as no nation survives independently; however, Africans have to take the centre stage in solving their problems. Malaria control and indeed a world free of malaria is achievable only if the core stakeholders, Africans step up their games. These stakeholders' roles must be taken seriously by all involved in order to achieve the vision 2030 of a world free of malaria.
